# Feature Refinement Method Based on the Two-Stage Detection Framework for Similar Pest Detection in the Field

**DOI:** 10.3390/insects14100819

**Published:** 2023-10-16

**Authors:** Hongbo Chen, Rujing Wang, Jianming Du, Tianjiao Chen, Haiyun Liu, Jie Zhang, Rui Li, Guotao Zhou

**Affiliations:** 1 Science Island Branch of Graduate School, University of Science and Technology of China, Hefei 230026, China; hbchen1@mail.ustc.edu.cn (H.C.); tjchen@mail.ustc.edu.cn (T.C.); liuhaiyun@mail.ustc.edu.cn (H.L.); zhangjie@iim.ac.cn (J.Z.); 2 Institute of Intelligent Machines, Hefei Institutes of Physical Science, Chinese Academy of Sciences, Hefei 230031, China; lirui@iim.ac.cn; 3 Institutes of Physical Science and Information Technology, Anhui University, Hefei 230039, China; 4 Henan Yunfei Technology Development Co., Ltd., Zhengzhou 450003, China; luchunguang@hnyfkj.cn

**Keywords:** pest detection, field environment, similar pests

## Abstract

**Simple Summary:**

The larvae of Lepidoptera pests are polyphagous insects that can cause crop mortality and severely damage crop growth, but the manual detection of such pests is a time-consuming and laborious task. We propose an automatic detection method to distinguish similar pests in the field. The proposed method is implemented based on the object detection framework, which improves the feature description ability of the network for different pests, optimizes suboptimal feature selection, and focuses the network head toward specific tasks. Our method achieves better detection results on a similar pest dataset compared with other advanced algorithms.

**Abstract:**

Efficient pest identification and control is critical for ensuring food safety. Therefore, automatic detection of pests has high practical value for Integrated Pest Management (IPM). However, complex field environments and the similarity in appearance among pests can pose a significant challenge to the accurate identification of pests. In this paper, a feature refinement method designed for similar pest detection in the field based on the two-stage detection framework is proposed. Firstly, we designed a context feature enhancement module to enhance the feature expression ability of the network for different pests. Secondly, the adaptive feature fusion network was proposed to avoid the suboptimal problem of feature selection on a single scale. Finally, we designed a novel task separation network with different fusion features constructed for the classification task and the localization task. Our method was evaluated on the proposed dataset of similar pests named SimilarPest5 and achieved a mean average precision (mAP) of 72.7%, which was better than other advanced object detection methods.

## 1. Introduction

Crop pests cause serious harm to crop growth [1]. Accurate detection of different pests and the implementation of corresponding control measures can effectively improve crop yield and quality, which are crucial for agricultural production. One such destructive insect pest is the fall armyworm, *Spodoptera frugiperda* (Smith), flagged as a major concern by the Food and Agriculture Organization of the United Nations (FAO) [2,3]. Field investigations have revealed that *S. frugiperda* can easily be confused with other lepidopteran insect pests, such as *Mythimna separate* (Walker), *Ostrinia furnacalis* (Guenee), *Helicoverpa armigera* (Hübner), and *Spodoptera litura* (Fabricius), as they are similar in size, color, morphology, and living environment, particularly in their larval stage [4]. The similarity of these visual features poses a challenge for pest identification. Incorrect identification results lead to the use of unsuitable pesticides, which may not only jeopardize the growth of crops but also cause environmental pollution. Traditional pest identification tasks are completed by agricultural plant protection personnel through manual surveys, which are inefficient and susceptible to subjective factors. Fortunately, recent advancements in computer vision technology have provided new ideas and technical support for the automatic detection of some insect pests, which can effectively reduce the workload of professional plant protection personnel [5].

Traditional computer vision algorithms mainly use image processing and pattern recognition techniques for the feature extraction and classification of pest images. A large number of studies [6,7,8] have been conducted to extract color, shape, and texture features of pests and perform pest recognition tasks using support vector machines (SVMs) with certain results. In real-world outdoor scenarios, complex background environments are often present. Additionally, pest images are susceptible to various factors, such as differing light intensities, diverse weather conditions, and crop obstructions. Therefore, pest detection algorithms based on traditional computer vision face certain difficulties in adapting to complex field environments.

Compared with traditional machine learning techniques, deep learning techniques can fit the intrinsic characteristics of a large number of data well with a higher accuracy rate and strong robustness [9]. The detection-based method calculates the location and species of multiple pests in an image, which can reduce the interference of a complex background when the size of pests is relatively small in the image. The field of object detection is mainly divided into single-stage detection algorithms, represented by the YOLO series [10,11,12,13,14,15], and two-stage detection algorithms, represented by Faster RCNN [16]. Many advanced algorithms have been derived, such as Retinanet [17], Cascade RCNN [18], Double Head [19], etc. To improve the detection ability of multi-scale objects, the feature pyramid network (FPN) [20] was added into these detection frameworks as a common component. Compared with the fast single-stage algorithm, the two-stage algorithm has a slower speed but achieves higher accuracy.

Recently, many researchers have introduced deep learning technology into the field of agriculture. Many researchers [21,22,23] have achieved high recognition results on cropped datasets by using classification methods for pest recognition. For the detection of dense small-sized pests in complex environments, Li and Teng et al. [24,25] designed a coarse-to-fine network and a pyramid network to enhance the detection effect. Many researchers [26,27] constructed databases by fixed pest collection devices and utilized detectors such as YOLOv5 for pest detection. In order to enhance the discrimination ability of detectors for multiple categories of pests, feature fusion [28,29] was considered for algorithmic improvement, and it was experimentally demonstrated that feature fusion is effective in improving detection accuracy. Classification-based methods [21,22,23] focused on the global features of images. However, the proportion of pest areas in images collected in the field is usually small, which leads to more invalid information contained in global features. Some of the methods mentioned above are optimized mainly for pest detection algorithms in specific scenarios [24,25,26,27], which are not applicable to similar pest detection in complex field environments. Although the improved feature fusion methods [28,29] can improve detection results, they are optimized only from the perspective of feature fusion and still have some limitations.

**Figure 1:**
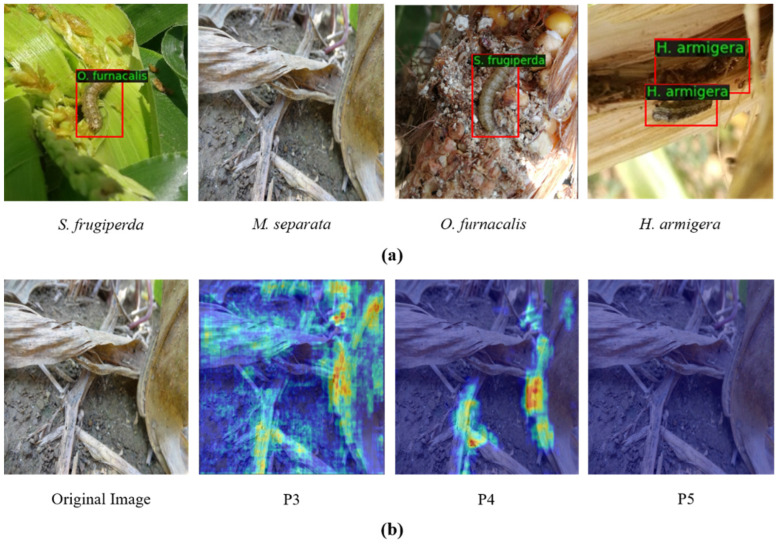
(**a**) Examples of detection results for similar pests in the field based on the Cascade RCNN algorithm. (**b**) Feature response maps of the pest image at different scales, where P3, P4, and P5 represent the feature response maps of the third, fourth, and fifth layers, respectively. The response of the pest region on the P5 layer feature map was not significant.

As shown in Figure 1a, pest identification problems arise from inaccurate detection and recognition. The main factors that affect the accuracy of field pest identification are as follows: (1) the complex living environment of field pests and the similarity in appearance among different species of pests, which can lead to confusion in the extraction of pest features; (2) the responses of pest regions are different on multiple-scale feature maps, and incorrect feature mapping results in missed detection of pests, as shown in Figure 1b; (3) the current two-stage pest detectors adopt the same RoI (region of interest) feature for the classification task and the localization task, which may make it difficult to train the task head network with optimal parameters; and (4) due to the difficulty of pest image acquisition in complex scenes, there is a lack of similar pest datasets for real farmland scenes.

To address the aforementioned issues, some methods based on feature enhancement [30], feature fusion [31], and feature separation [19] were proposed. Inspired by this, we optimized the two-stage algorithm in terms of contextual information, adaptive feature fusion, and separating features for diverse tasks to further improve the pest detection accuracy. Firstly, a context feature enhancement module was constructed to generate multi-scale features, which were used to enhance the features extracted by the backbone network. Secondly, the attention mechanism was used to adaptively weight the fusion of pest RoI features on the multi-scale feature map to obtain more accurate features of the pest regions. Finally, different features were constructed for separating the classification and localization tasks. The multi-scale features extracted by the context feature enhancement module were used for the localization task, and the context-enhanced features fused by the FPN were used for the classification task.

The main contributions of our work can be summarized as follows:

(1) A context feature enhancement module (CFEM) was proposed to obtain attention maps at each scale by atrous spatial pyramid pooling, which was beneficial for the detection of similar pests.
(2) We proposed the RoI feature fusion module (RFFM) to adaptively weight and fuse pest features on multiple network layers, which was more conducive to the classification and localization of pests at different scales.
(3) The proposed task separation module (TSM) decoupled the features of pest classification and localization networks, improving the overall performance of the detector.
(4) A larval dataset, SimilarPest5, containing five similar pest species was established to demonstrate the effectiveness of the developed method.

## 2. Materials and Methods

### 2.1. Dataset

In recent years, several pest datasets have been published [32,33] which contain various species of pests but have either low similarity between pests or a small number of samples for each pest category. In addition, the image backgrounds in some datasets are homogeneous and significantly differ from the actual field environment. To achieve the specific task of accurately identifying similar pests in the field, we constructed a dataset named SimilarPest5, consisting of 5177 images, all of which were collected from the field environment. A comparison of multiple insect pest datasets is shown in Table 1.

The feeding habits of most Lepidoptera pests are significantly different between larval and adult stages. The larvae mainly feed on the leaves, stems, and ears of crops, causing serious damage to their growth [2]. The SimilarPest5 dataset mainly collects the larvae of five similar pests, including *S. frugiperda*, *M. separata*, *O. furnacalis*, *H. armigera*, and *S. litura*. The images in the SimilarPest5 dataset were collected in five different cities and counties in China, and the collection period was mainly focused on the period from July to October between 2020 and 2022. Weather conditions were mostly sunny or cloudy during the acquisition process to ensure the clarity and visibility of the images. The crop species in the images are mainly maize, and a small number of images from soybean fields are also included. To increase the generalization capability of the dataset, we used different kinds of acquisition devices, such as digital cameras and smartphones from different manufacturers. In addition, we acquired images from multiple angles and distances to obtain more visual information while ensuring that the pests were clearly visible. This diverse data collection approach contributed to a comprehensive and diverse collection of field pest images. To minimize crop damage, some obstructive objects were removed to ensure the capture of larvae on the stems and leaves. The SimilarPest5 dataset contains only similar pests in complex environments in the field, and the number of images for each pest reaches about 1000, which is different from other pest datasets. Different species of pests have similar morphology and appearance, which poses significant challenges for precise classification and localization. In addition, the complex field environment causes interference, such as obstruction and lighting, in some pest images.

We uniformly scaled the image width and height to 800 × 600 pixels and used LabelImg (https://github.com/tzutalin/labelImg) (accessed on 28 March 2023) software to annotate the pests in the images. We invited researchers from the Academy of Agricultural Sciences and agricultural experts to annotate the pest images. To ensure the accuracy of annotation, each expert focused on only one pest species. Finally, all experts collaborated to check the correctness of each annotation instance. Annotation information mainly included pest ID and location coordinates, which were stored in XML format. For training detection models, these collected pest images were divided into a training set (80%) and a testing set (20%). Table 2 reports the statistical data for each pest species.

Table 3 provides the statistical data of pests at each scale. According to the division standard of the MS COCO [34], objects smaller than 32 × 32 pixels are defined as small objects, those from 32 × 32 to 96 × 96 are considered medium, and those greater than 96 × 96 are defined as large objects. The sample scale distribution in SimilarPest5 is mainly concentrated in the range of medium and large objects.

**Figure 2:**
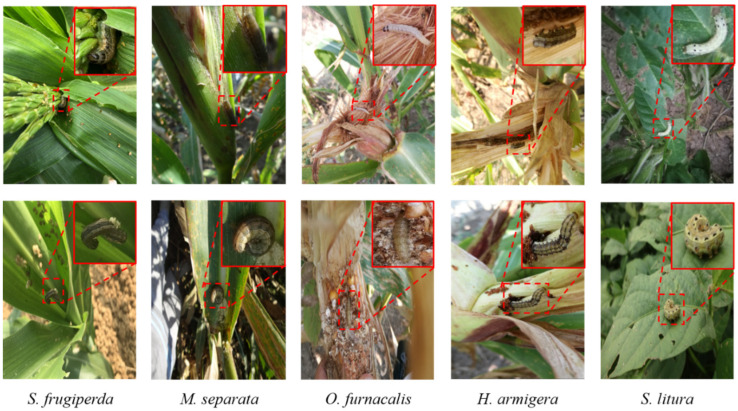
Examples of various pest images from the SimilarPest5 dataset. The pests in SimilarPest5 have a high similarity of color, texture, shape, and living environment.

The larval images of the target insect pests in the SimilarPest5 dataset are shown in Figure 2. Different pest species have similar morphology and appearance. Additionally, due to the living habits of pests in the field, some pests in the images are subject to interference, such as occlusion and lighting, which weakens the feature information used to distinguish between different species of pests and between foreground/background. All these factors pose significant challenges to the accurate classification and positioning of pests.

### 2.2. Methodologies

**Figure 3:**
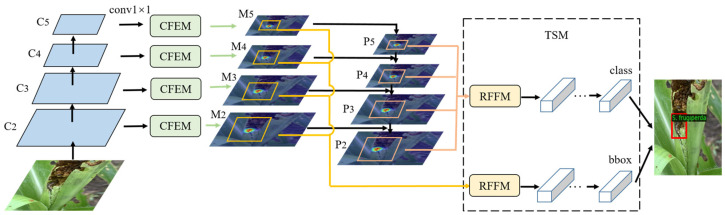
Overall architecture of our two-stage pest detector. The CFEM is used to enhance features at each scale, and the RFFM is used to fuse the RoI features of a multi-scale pyramid. The TSM constructs independent feature and task head networks for classification and localization.

In general, the detection speed of the first-stage detector is faster, but the detection accuracy is not higher than that of the two-stage detector [35]. Therefore, we focused on studying the feature refinement method based on the two-stage detection framework to improve the accuracy of pest detection, and the Cascade RCNN [18] algorithm was used as the baseline network. First, pest images were fed into a backbone network to extract features. Then, the extracted feature maps were fed into the feature enhancement module (CFEM) to generate high-quality enhanced features. Next, after the enhanced feature maps were processed through the FPN, the RoI feature fusion module (RFFM) fused target region features at multiple scales. Finally, the task separation module (TSM) decoupled the features of different tasks to achieve the pest classification and localization. The overall framework of the pest detector is shown in Figure 3, and a detailed description of the modules is given in the following subsections.

#### 2.2.1. Context Feature Enhancement Module

**Figure 4:**
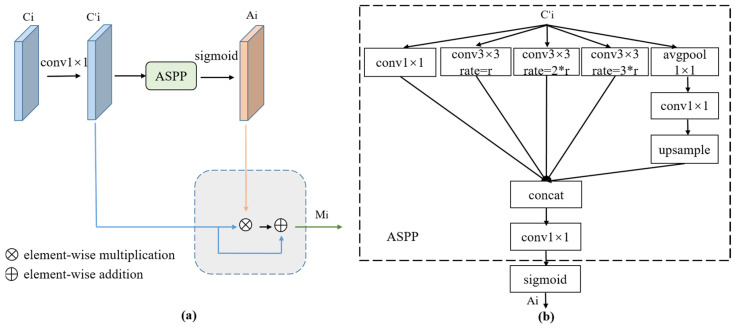
(**a**) Architecture of the CFEM, where *C_i_* denotes the $i^{th}$ layer features extracted by the backbone network. (**b**) Detailed network structure of $C_{i}^{\prime }$ to $A_{i}$, where ‘*r*’ represents the sampling rate.

In order to enhance the feature description ability of the network for the target pests, we designed the CFEM to generate enhanced features of different layers. Different from the global feature enhancement based on the backbone network [30], the CFEM captured contextual information using multi-scale receptive fields, which helped the model understand semantic information of an image at different scales. As shown in Figure 4, a 1 × 1 convolution operation was conducted on each scale of features, Ci, extracted from the backbone network to ensure a uniform number of channels for each scale feature. In this paper, the number of channels was 256. In order to obtain the multi-scale context information of the target pest, atrous spatial pyramid pooling (ASPP) [36] with multiple sampling rates and effective target field of view was employed to generate the context information for the corresponding scale layers. The context heat map of the corresponding layers was obtained through the sigmoid activation function. The contextual feature maps of the different layers have differing scale biases. To enhance the information of the specific scale object, the contextual features of the corresponding layers were added to the original features using residual connections to avoid the pest features at specific scales being overwhelmed by background information.

The whole computation process can be summarized as follows:

(1) $$A_{i}=\sigma (f_{aspp}(\varphi_{i}(C_{i}))),$$

(2) $$M_{i}=(1⊕A_{i})⊗\varphi_{i}(C_{i}),$$

where σ is the sigmoid activation function, $\varphi_{i}$ denotes a 1 × 1 convolution operation at the $i^{th}$ layer, $f_{aspp}$ indicates the ASPP context-aware operation, and $A_{i}$ denotes the context features at the $i^{th}$ layer. $C_{i}$ represents the output features of the $i^{th}$ layer of the backbone network, $M_{i}$ represents the enhancement features of the $i^{th}$ layer, ⊕ denotes element-wise addition, and ⊗ denotes element-wise multiplication.

#### 2.2.2. RoI Feature Fusion Module

In the feature pyramid structure, high-resolution feature maps have more detail and are more sensitive to small objects, while low-resolution feature maps have a high degree of semantic information and are usually employed in the detection of large objects [20]. The general two-stage object detection algorithm maps the feature of the proposal box to a specific layer of the FPN by the size of the proposal box to obtain the RoI features. However, this approach may lead to incorrect detection results because the proposal boxes of the target may not be assigned to the optimal feature map.

**Figure 5:**
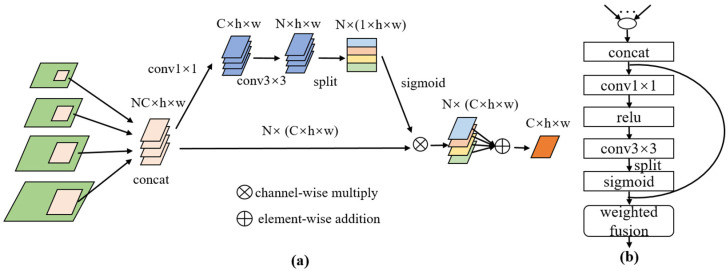
(**a**) Architecture of the RFFM. “N” represents the number of RoI feature maps, which is 4 in this paper. “C” represents the channel number of RoI features, which is 256 in this paper. (**b**) Detailed network structure of the RFFM.

The effectiveness of the attention mechanism in feature fusion has been verified, and representative algorithms include channel attention networks and spatial attention networks, such as SENet [37] and CBAM [38]. The attention module can learn weight parameters adaptively, instead of mapping proposal boxes to one feature map. Unlike PANet [31], which used a fully connected layer to fuse all pyramid-level RoI features, we adopted the RFFM to adaptively aggregate the RoI features of the different scales from all feature maps. As shown in Figure 5, RoIAlign [39] was used to extract the RoI features of the P2–P5 layers, with a feature size of 7 × 7, and then these features were connected. To reduce the computational effort, the RFFM module initially performed feature dimensionality reduction. It subsequently adaptively calculated the weights of the features at each scale and finally performed weighted fusion of the features. After multiple convolutional operations and the sigmoid activation function, the spatial weights of multiple layers were obtained. The RoI features of different layers were weighted and fused with the weights of the corresponding layers to obtain the final fused features. It is important to note that the weight parameters were adaptively learned with the back-propagation of the network, which avoided the hard selection of RoI features and achieved better detection results.

The RFFM formula is expressed as follows:

(3) $$w=\sigma (\phi^{3}(\psi (\phi^{1}(R_{c}))),$$

(4) $$R=\sum_{i=2}^{5}w_{i}⊗R_{i},$$

where $R_{c}$ denotes the RoI features after the concatenate operation; $\phi^{1}$ and $\phi^{3}$ denote the 1 × 1 convolution and 3 × 3 convolution, respectively; ψ is the ReLU activation function; σ is the sigmoid activation function; w is the adaptive weight of the RoI features; $w_{i}$ is the weight of the $i^{th}$ layer after splitting; $R_{i}$ represents the RoI features of the feature pyramid at the $i^{th}$ layer; and R is the features after adaptive fusion.

#### 2.2.3. Task Separation Module

High-level semantic information is helpful for classification, while localization is more sensitive to details [40]. Therefore, the features suitable for classification and localization may not always be consistent. The Double Head [19] algorithm was decoupled from the localization head and the classification head, which leads to better performance. However, they still share the same RoI feature.

**Figure 6:**
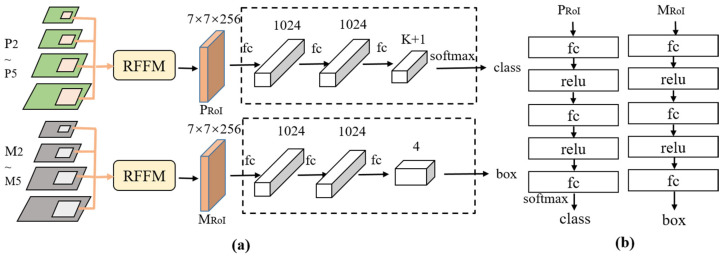
(**a**) Architecture of the task separation module. “K + 1” represents the number of pest categories and the background. “PRoI” and “MRoI” denote the adaptive fusion features of candidate regions on different feature maps. (**b**) Detailed network structure of the classification and localization network.

In this study, we focused on constructing different features for pest classification and localization tasks. Notably, the fusion features within layers P2 to P5, connected from top to bottom, have enhanced semantic information. Conversely, the fusion features in the layers M2 to M5 have richer detail information. Therefore, the RoI features extracted by the RFFM on the FPN output features (P2–P5) were used for the classification task, while the RoI features extracted by the RFFM on the CFEM output features (M2–M5) were used for the localization task. As shown in Figure 6, the first stage of the two-stage object detection algorithm would output proposal boxes, which were mapped to the M2–M5 and P2–P5 feature maps. The RoI features on M2–M5 and P2–P5 were adaptively fused by their corresponding RFFMs. Then, the output features were independently used for localization and classification tasks through separate task branches while maintaining feature consistency. For the classification task, we employed the cross-entropy loss function, and for the localization task, the Smooth L1 loss function was used.

#### 2.2.4. Parameter Settings

All the experiments were based on the SimilarPest5 dataset. In our experiments, the ResNet-50 [41] and ConvNext-B [42] models trained on the ImageNet [43] dataset were used as pre-trained models. The size of the input images to the network was proportionally adjusted to (1333, 800). The experiments were based on single-scale training and testing of the MMDetection [44] object detection framework. The experiments were conducted on the operating system Ubuntu 18.04 based on Python 3.7, PyTorch 1.10, and CUDA 11.3. In our experiments, two NVIDIA TITAN RTX GPUs with 24 GB of memory were used. All experiments were iteratively fine-tuned for 12 epochs, and the optimizer SGD (stochastic gradient descent) was adopted to train the models. The learning rate was initialized to 0.005 and reduced to one-tenth after the 8th and 11th epochs. The hyper-parameter settings are shown in Table 4, and other parameters were set to the defaults of MMDetection [44]. Due to memory constraints, all layers were fine-tuned with a stochastic gradient descent (SGD) optimizer in mini batches of size 2. The random flip operation was used in the training phase with a random scale of 0.5.

## 3. Results

### 3.1. Evaluation Metrics

The evaluation metric is an important basis for evaluating the performance of a method. To ensure the fairness of an experimental comparison, the standard evaluation metrics for the general object detection task are used. These metrics use the intersection over union (IoU) to represent accuracy in predicting bounding boxes and evaluate the performance. The average precision (AP) indicates the detection performance of each category; it is the area bounded by the precision-recall curve. The mean average precision (mAP) was used to evaluate the overall performance, and it represented the mean value of the AP for all categories, starting from 0.5 for the IoU threshold and increasing by steps of 0.05 up to 0.95. The mean recall (mRecall) represented the mean value of the recall for all categories, starting from 0.5 for the IoU threshold and increasing by steps of 0.05 up to 0.95. The calculation formulas are as follows:

(5) $$Precision=\frac{TP}{TP+FP}$$

(6) $$Recall=\frac{TP}{TP+FN}$$

(7) $$AP=\int_{0}^{1}P(R)d(R)$$

where TP, FP, and FN denote true positives, false positives, and false negatives, respectively.

### 3.2. Comparison with State-of-the-Art Methods

To illustrate the overall performance of the proposed method, we conducted a comparison with other advanced object detection methods, including CNN-based one-stage methods (RetinaNet [17], YOLOF [45], YOLOV5 [13], and YOLOV8 [14]) and two-stage methods (Faster RCNN [16], Double Head [19], Libra RCNN [46], Cascade RCNN [18], and Sparse RCNN [47]), as well as the transformer-based object detectors (such as Dino [48]). Table 5 reports the experimental results for the SimilarPest5 dataset, and the training process of the model was reported in the Supplemental Materials (Figures S1 and S2). The proposed modules were integrated into the Cascade RCNN [18] framework. Compared with the other methods, our proposed method achieved the highest mAP of 72.7%, 1.2% higher than the other best-performing algorithm. Additionally, the proposed method performed better in the detection of medium- and large-scale pests with a higher recall rate. This indicated that feature optimization modules can improve the detection accuracy for similar pests in the field. As additional modules were added to the original two-stage detection framework, the overall number of parameters of the proposed method increased, resulting in a decrease in FPS (frames per second). Table S1 of the Supplementary Material reports the AP of different methods for detecting each category based on the ConvNext-B [42] backbone network.

**Figure 7:**
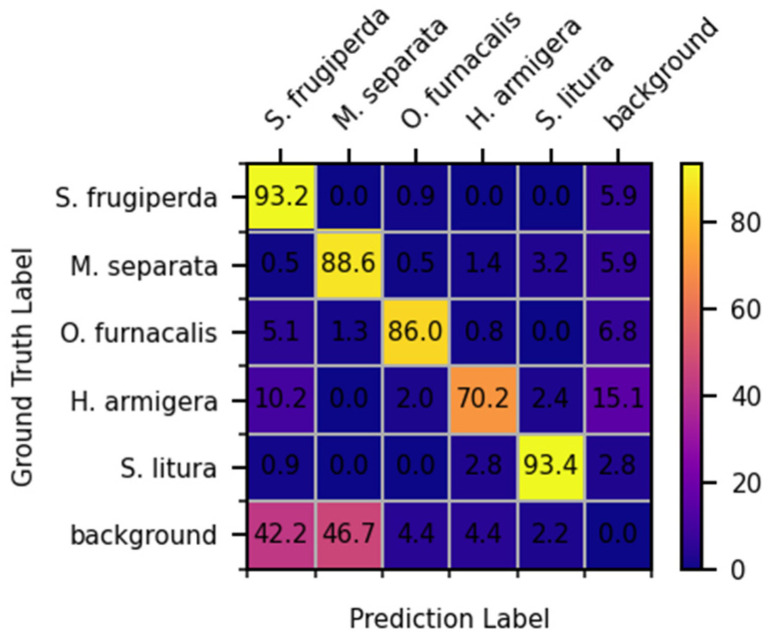
Confusion matrix of the proposed method with a confidence threshold equal to 0.5 (unit: %).

Figure 7 shows the confusion matrix of the proposed method when the confidence threshold was equal to 0.5. The diagonal represents the percentage of each pest that was correctly identified. The complex field environment is the main reason for the misidentification between pest targets and backgrounds. The similarity in appearance led to confusing identifications between pests. As shown in Figure 7, the accuracy for *S. frugiperda* was reduced because some of *H. armigera*, *O. furnacalis*, and the background were misidentified as *S. frugiperda*. A larger proportion of *H. armigera* was misidentified as *S. frugiperda* and the background, which reduced the accuracy for *H. armigera*. In addition, there were different proportions of confusing identifications for *M. separata*, *O. furnacalis*, and other pests. The accuracy for *S. litura* was higher due to the lower percentage of misidentifications.

### 3.3. Ablation Experiments

**Figure 8:**
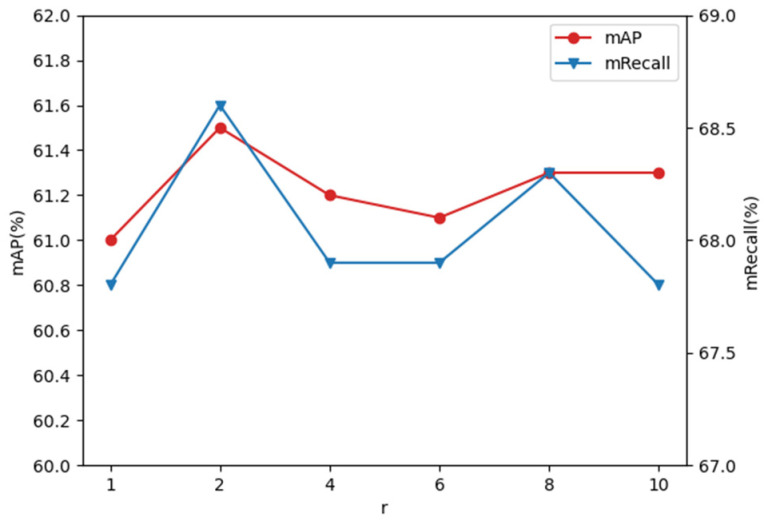
Detection accuracy with change in the sampling rate, *r*.

We conducted several experiments on the SimilarPest5 dataset to explore the effect of the sampling rate, *r*, on detection accuracy in the ASPP network. The detection results were evaluated by the metrics of mRecall and mAP, as shown in Figure 8. For efficiency, the experiments were conducted using the Faster RCNN detector with our constructed CFEM by using ResNet-50 [41] as the backbone. When *r* equaled 1, the pest features were not sufficiently correlated with the surrounding contextual information to achieve optimal accuracy. Too large an *r* led to a higher correlation of pest features with distant background information, decreasing accuracy. Therefore, setting *r* to 2 was more suitable for our dataset, and the mAP and mRecall achieved the highest accuracy.

**Figure 9:**
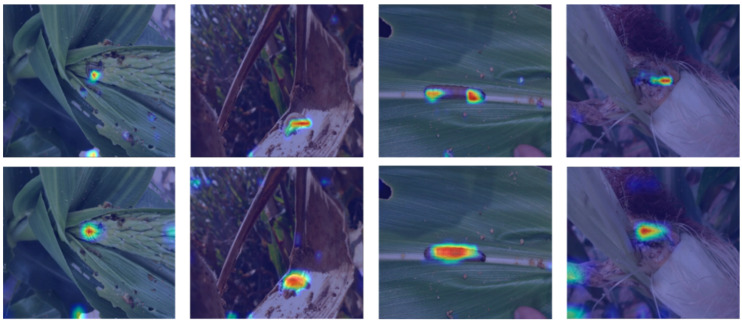
Comparison of feature response maps. The first row shows the feature response maps of the original pest features fused by the FPN, and the second row shows the feature response maps after adding the CFEM. All feature response maps were from the P4 layer, and ResNet-50 was used as the backbone network.

To verify the effectiveness of the CFEM in enhancing pest feature expression, we compared the feature response maps before and after adding the CFEM based on the Faster RCNN [16] algorithm framework in Figure 9. The features enhanced by the CFEM had stronger semantic correlation between local and contextual features of pests. The incorporation of richer contextual information made the feature responses of pest regions more obvious and accurate. Figure S3 of the Supplementary Material shows the feature response maps of our method to different pests at multiple scales.

Based on the Faster R-CNN [16] framework, we constructed ablation experiments with different features selected for the classification task and the localization task of the TSM. The results of these experiments are shown in Table 6. From the results, we could observe that the highest detection accuracy of 63.2% mAP was obtained when using M2~M5 layer features for the localization task and P2~P5 feature layers for the classification task. These results indicated that M2~M5 layer features were more suitable for localization, while P2~P5 layer features fused by the top-down pathway had stronger semantic information and were more suitable for pest classification.

To further validate the effectiveness of each module, we constructed ablation experiments for each module based on the Faster RCNN [16] framework. As shown in Table 7, the addition of any module led to a performance improvement. As shown in the second row of the table, the CFEM could effectively enhance the feature representation ability and achieved a 1.4% mAP improvement. The improvement shown in the third row indicated that the RFFM adaptively fusing multi-layer RoI features outperformed the method with single-layer RoI features in accuracy. The fourth row shows the result of introducing the TSM with a 0.3% mAP improvement, which indicated that the decoupling of tasks based on different features had a positive effect on the detection accuracy improvement. Finally, the proposed method achieved a 63.2% mAP, which was a 3.1% improvement over the original method, and was accompanied by a higher recall rate.

As the proposed method can be embedded as a plug-and-play plugin into most existing two-stage object detection frameworks, we conducted experiments to verify the applicability of the proposed modules with different methods. As shown in Table 8, the mAP of the three methods improved by 3.1%, 2.2%, and 1.6%, respectively, with the addition of our module, and the recall rate also increased. The experimental results demonstrated that the proposed feature refinement modules had good generalization ability.

### 3.4. Visualization Analysis

**Figure 10:**
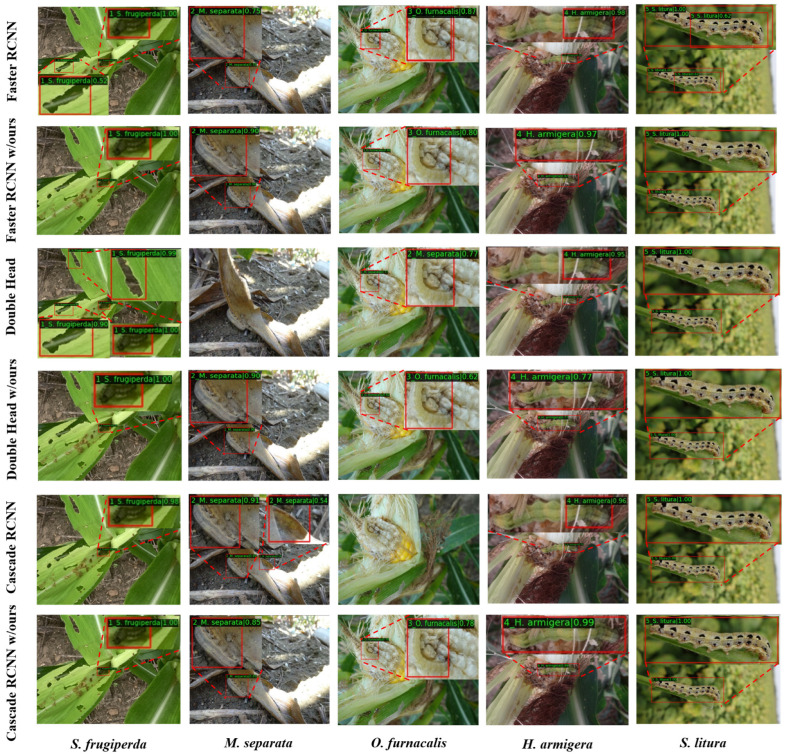
Visualization of the detection results of the two-stage detection methods before and after optimization. The second, fourth, and sixth rows show the detection result images with the proposed modules added on different method frameworks.

**Figure 11:**
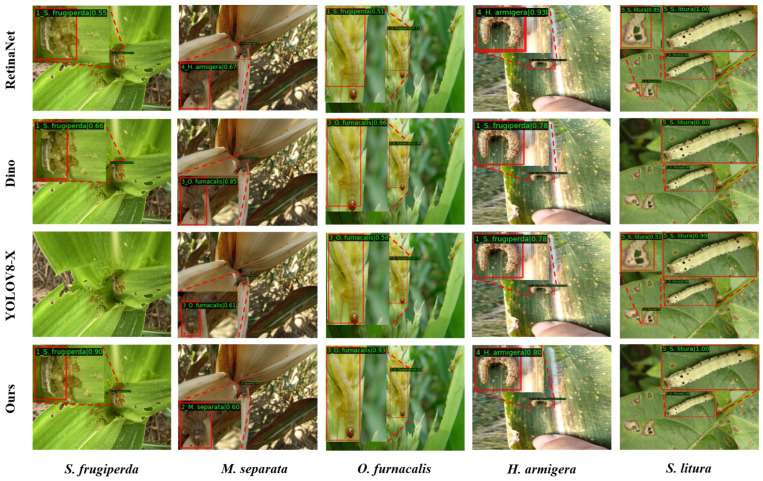
Visualization of the detection results of our method compared with other detectors.

The visualization results provide us with a more intuitive way to observe the performance improvement of the proposed method. In Figure 10, we compared the detection results of some two-stage detection methods before and after optimization. Due to the similarity in color and texture between some pests and the complex background, such as *M. separata* and *O. furnacalis*, this resulted in missed detections with Double Head [19] and Cascade RCNN [18]. For the detection of *S. frugiperda*, Faster RCNN [16] and Double Head [19] incorrectly recognized objects in the background as the target pest. Additionally, Double Head [19] showed category confusion in identifying *O. furnacalis*. Although these methods were able to correctly recognize pests in detecting *H. armigera* and *S. litura*, they were not accurate enough in pest localization due to partial occlusion of the pest ontology in some training image sets. In Figure 11, we compared the detection results of the proposed method with those of the other methods. Some other methods also showed incorrect identification results, e.g., Dino [48] and YOLOV8-X [14] misidentified *H. armigera* as *S. frugiperda*, while RetinaNet [17] and YOLOV8-X [14] misidentified the background as *S. litura*. By comparing the detection results of different methods, it can be found that our method is more accurate in both pest classification and localization.

### 3.5. Discussion

Traditional computer vision algorithms are simple in design and low in computational resource consumption, but weak in feature representation for complex scenes. Therefore, many researchers have started to focus on the application of deep learning methods for pest identification. However, classification-based methods [21,22,23] usually require tedious preprocessing processes, such as manually cropping or segmenting out pest regions, while the algorithms themselves focus mainly on the classification task and have limited applications. In images of field pest datasets, the size and location of pests vary widely, and thus classification-based methods are not adapted to datasets in field environments. In pest identification methods based on object detection frameworks [24,25,26,27,28,29], researchers have designed specific algorithmic frameworks for the characteristics of different pest datasets or optimized them only from the perspective of feature fusion. However, the pests in these datasets differ significantly from the SimilarPest5 dataset in terms of scale, context, and similarity.

Although generic object detection algorithms perform well in most tasks, they often struggle to achieve outstanding results in specific tasks. From the results shown in Table 5, the detection accuracy of two-stage algorithms [16,18,19,46] is usually higher compared to single-stage algorithms [14,17,45] on similar pest datasets. Since two-stage algorithms achieve detection through a coarse-to-fine process of object classification and localization, they are more suitable for fine-grained tasks such as the detection of similar pests in the field.

In this study, we designed a feature refinement method based on the two-stage detection framework with the aim of improving the detection accuracy for similar pests in the field. The two-stage algorithm was optimized by adding feature enhancement, feature fusion, and feature selection modules, and the overall detection accuracy of this method reached 72.7% mAP. Although the proposed method achieved the best accuracy, the subtle differences between the same types of pests at different age stages, the similar appearances between different species of pests, and complex background environments posed challenges for the detection algorithm. As the *S. litura* sample contained some images of soybeans that had different backgrounds from the images of corn, this led to the highest detection accuracy for *S. litura*, while similar pests from the same crop were more likely to be confused.

To verify the effectiveness of the proposed method, several ablation experiments were constructed. As shown in Figure 9, the semantic correlation of pest region features was stronger due to the fusion of contextual information at multiple scales, and the richer feature information helped to distinguish different pests. The ablation experiment in Table 7 showed that the RFFM of adaptive fusion of multi-scale region features could improve the detection accuracy compared with selecting only single-scale region features. Furthermore, the fourth and sixth rows of Table 7 verified that feature separation of different tasks allowed different task heads to focus more on specific tasks, thereby enabling the network to train better parameters to improve accuracy. Table 8 showed the generality of the proposed method on different models, with improved accuracy for these models. Overall, compared with other excellent detection algorithms, the proposed method based on the Cascaded RCNN [18] framework was superior in terms of overall detection accuracy.

## 4. Conclusions

In agricultural production, many lepidopteran pests with similar appearances, represented by *S. frugiperda*, cause serious damage to crop growth. Therefore, we constructed a SimilarPest5 dataset with images of five similar pests in corn and soybean fields. However, since these images were obtained in actual corn and soybean field environments, they frequently exhibit challenges like complex backgrounds, uneven lighting, and occasional obstruction. These factors make it challenging for a generic model to describe the features of the pests accurately. To improve the accuracy of pest detection in real-field scenarios, we optimized the detection network from different perspectives. The effectiveness of the proposed modules was validated through ablation and comparison experiments, and state-of-the-art performance was achieved on the SimilarPest5 dataset.

The method proposed in this paper can provide intelligent recognition functions for terminal devices and reduce the burden on professionals (Figure S4). In real-world IPM scenarios, higher pest detection accuracy can provide effective decision support for pest control, while providing early warning information to agricultural personnel and helping to develop more effective IPM strategies. However, our proposed method still has some limitations. In future work, we hope to collect and construct larger datasets of field pests from different crops and extend the proposed method to other types of pest detection, such as rice pests like *Nilaparvata lugens Stal*, *Sogatella furcifera*, and *ladelphax striatellus falln*, to explore the effectiveness of the method in pest detection for other crops. Furthermore, we would like to further analyze the similarity of pests based on their morphological, ecological, and statistical characteristics and verify the detection accuracy of the proposed algorithm for pests with different similarity levels. Since the algorithm introduces additional modules, it leads to greater computational complexity and requires support from hardware devices with higher computational performance. Therefore, in future work, we will try to build lighter model architectures while maintaining detection performance.

## Figures and Tables

**Table 1:** Comparison of existing datasets related to insect pests. “Class” denotes the class number. “Samples number” represents the number of images. “Crop” refers to the crop species involved in the acquisition of pest images. “Stage” indicates the developmental stage of the pests. “Avail” indicates if the dataset is available. “Y” and “N” denote “yes” and “no”, respectively.

Dataset	Image	Class	Samples Number	Crop	Stage	Avail	Similarity
Tetila et al. [21]	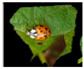	13	5000	Soybean	Larvae	N	Low
Li et al. [24]	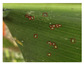	1	2200	Wheat, rapeseed	Larvae, adult	N	/
Jiao et al. [29]	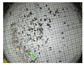	21	2442	Fixed equipment	Adult	N	Low
Wang et al. [32]	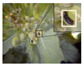	14	49,707	Rice, wheat, maize, rapeseed	Larvae, adult	N	Low
IP102 [33]	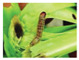	102	75,222	Rice, wheat, mango, etc.	Larvae, adult	Y	Low
SimilarPest5	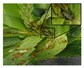	5	5177	Maize, soybean	Larvae	/	High

**Table 2:** Statistics of the SimilarPest5 dataset. “Crop” refers to the crop species involved in the acquisition of pest images. “Samples number” indicates the number of pest images. “Instances number” indicates the number of pest targets in all images. “Training set” indicates the number of images in the training set. “Test set” indicates the number of images in the test set.

Pest ID	Categories	Samples Number	Instances Number	Training Set	Test Set
1	*S. frugiperda*	1071	1152	857	214
2	*M. separata*	1023	1141	819	204
3	*O. furnacalis*	1038	1070	831	207
4	*H. armigera*	1038	1041	832	206
5	*S. litura*	1007	1025	806	201

**Table 3:** Statistics of pests at each scale in the SimilarPest5 dataset. “Ratio” indicates the number of pest instances at the corresponding scale as a proportion of the number of all pests.

Scale	Number	Ratio	Average Pixel
Medium	2149	39.6%	5304
Large	3280	60.4%	21,869

**Table 4:** Training hyper-parameters.

Batch Size	GPUs	Epoch	Optimizer	Learning Rate	Weight Decay	Momentum
2	2	12	SGD	0.005	0.0001	0.9

**Table 5:** Comparison experiments with other advanced object detection algorithms (unit: %). “Params” indicates the number of parameters (unit: M). “FPS” represents the number of frames processed per second.

Method	Backbone	*mAP*	*AP* *_M_*	*AP* *L*	*mRecall*	*Params*(*M*)	FPS
RetinaNet [17]	ResNet50	56.9	48.8	60.0	66.4	36.29	33.4
YOLOF [45]	ResNet50	62.8	55.2	65.9	70.4	42.27	52.6
Faster RCNN [16]	ResNet50	60.1	51.9	63.4	67.1	41.17	32.3
Libra RCNN [46]	ResNet50	62.4	52.4	66.2	70.4	41.43	30.8
Double Head [19]	ResNet50	63.3	52.9	67.2	70.0	46.76	14.2
Cascade RCNN [18]	ResNet50	64.5	50.7	69.1	70.8	68.95	26.8
Sparse RCNN [47]	ResNet50	63.0	52.4	67.4	74.1	105.95	25.6
Dino [48]	ResNet50	64.3	52.0	69.6	70.6	47.59	17.3
Ours	ResNet50	66.1	55.7	70.2	72.2	78.52	10.3
RetinaNet [17]	ConvNext-B	65.5	58.9	68.4	72.8	97.65	15.1
YOLOF [45]	ConvNext-B	66.2	58.7	69.8	73.5	105.97	18.5
Faster RCNN [16]	ConvNext-B	69.3	60.5	72.8	74.0	104.94	14.8
Libra RCNN [46]	ConvNext-B	69.9	61.3	73.3	75.0	105.20	14.5
Double Head [19]	ConvNext-B	71.4	61.8	75.1	76.1	110.52	9.3
Cascade RCNN [18]	ConvNext-B	71.3	60.9	75.4	76.2	132.73	13.7
Sparse RCNN [47]	ConvNext-B	67.9	58.8	71.9	74.2	169.74	13.8
Dino [48]	ConvNext-B	71.5	61.2	75.8	76.3	111.62	9.8
YOLOV5-L [13]	CSPDarknet	67.0	56.7	70.7	73.4	46.16	63.9
YOLOV5-X [13]	CSPDarknet	67.9	57.2	71.2	74.0	86.25	43.7
YOLOV8-X [14]	CSPDarknet	67.2	55.2	70.1	73.5	68.15	59.8
Ours	ConvNext-B	72.7	62.4	76.3	76.7	143.28	6.2

**Table 6:** Ablation studies with different features were selected for the classification task and the localization task of the TSM (unit: %).

Classification	Localization	*mAP*	*mRecall*
M2~M5	M2~M5	61.0	67.8
P2~P5	P2~P5	62.6	69.2
M2~M5	P2~P5	62.2	68.6
P2~P5	M2~M5	63.2	69.3

**Table 7:** Ablation experiments based on the Faster RCNN algorithm (unit: %). “Params” indicates the number of parameters (unit: M).

CFEM	RFFM	TSM	*mAP*	*mRecall*	*Params*(*M*)
			60.1	67.1	41.17
✓			61.5 (+1.4)	68.6 (+1.5)	50.06
	✓		61.9 (+1.8)	69.0 (+1.9)	41.41
	✓	✓	62.2 (+2.1)	68.7 (+1.6)	41.69
✓	✓		62.6 (+2.5)	69.2 (+2.1)	50.33
✓	✓	✓	63.2 (+3.1)	69.3 (+2.2)	50.61

**Table 8:** The performance of various detection methods with or without our module (unit: %).

Method	w/ours	*mAP*	*mRecall*
Faster RCNN [16]		60.1	67.1
	✓	63.2 (+3.1)	69.3 (+2.2)
Double Head [19]		63.3	70.0
	✓	65.5 (+2.2)	71.1 (+1.1)
Cascade RCNN [18]		64.5	70.8
	✓	66.1 (+1.6)	72.2 (+1.4)

## Data Availability

The original contributions presented in the study are included in the article/Supplementary Materials; further inquiries can be directed to the corresponding authors.

## Supplementary Materials

The following supporting information can be downloaded at: https://www.mdpi.com/article/10.3390/insects14100819/s1, Figure S1, Loss function curves of the proposed method based on different backbone network models; Figure S2, Comparison of the mAP of the proposed method with different “max_epochs” settings; Figure S3, Feature response maps of different pest images at multiple scales; Figure S4, Pest Identification System Client Application. Table S1. Performance of different methods as indicated by the detection results for each pest (unit: %).
