# Supplementary material for: Feature Refinement Method Based on the Two-Stage Detection Framework for Similar Pest Detection in the Field
Source: Insects. 2023 Oct 16;14(10):819. doi: 10.3390/insects14100819 (PMC10607060; doi:10.3390/insects14100819)
Supplement: Supplementary file 1 [file insects-14-00819-s001.zip › insects-2609648-supplementary.pdf]

Supplementary Materials

This document presents supplementary tables and figures for a paper entitled "**Feature Refinement Method based on the Two-Stage Detection Framework for Similar Pest Detection in the Field**".

Loss function curves and "max_epochs" settings

The training processes of Cascade RCNN and our model were compared in Figure S1. As the number of training iterations increased, the losses of the two models gradually decreased, and they tend to converge after two learning rate declines.

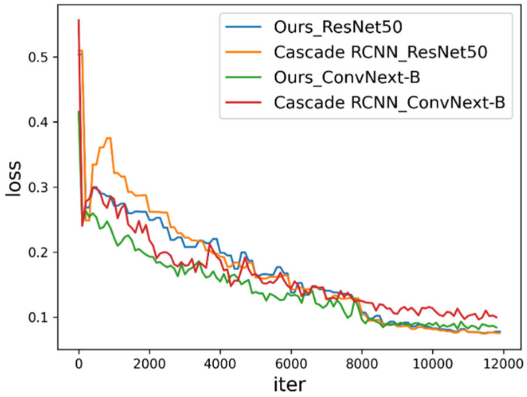

Figure S1. Loss function curves of the proposed method based on different backbone network models.

Figure S2 displayed the mAP of our algorithm at various epochs. With "max_epochs" set to 12, the learning rate was reduced to one-tenth after the 8th and 11th epochs. Similarly, with "max_epochs" configured at 36, the learning rate was decreased following the 27th and 33rd epochs. It was noticeable that the mAP stabilized after the 8th epoch, and there was an enhancement in mAP as the learning rate was decreased.

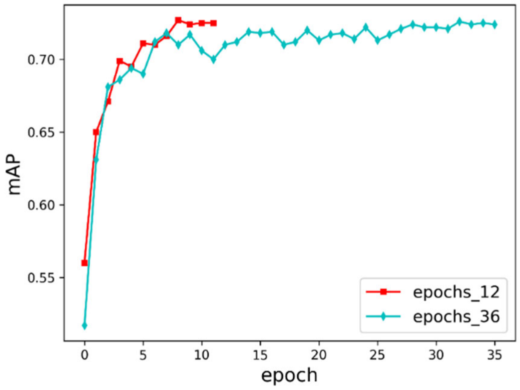

Figure S2. Comparison of the mAP of the proposed method with different "max_epochs" settings.

Detection results for each pest

Table S1 reports the detection results for each pest. The proposed method performed well on *S. frugiperda*, *H. armigera*, and *O. furnacalis*. For *M. separata* and *S. litura*, the accuracy obtained by the

proposed method was slightly lower than the other optimal methods. This may be due to the fact that the decoupling structure of Double Head [19] was more sensitive to the distribution characteristics of *M. separata* in our dataset, while the data-augmented and optimized YOLOV8-X [14] had better discriminative ability on *S. litura* from two different crops. However, they performed poorly on other pest categories, while our method performed better on pest species with higher similarity.

Table S1. Performance of different methods as indicated by the detection results for each pest (unit: %).

Method	*S. frugiperda*	*M. separata*	*O. furnacalis*	*H. armiger*	*S. litura*
RetinaNet [17]	55.6	70.3	60.6	65.8	75.2
YOLOF [45]	58.2	73.1	52.4	66.8	80.6
Faster RCNN [16]	61.1	75.5	64.4	65.7	79.8
Libra RCNN [46]	61.6	74.3	66.6	65.9	81.0
Double Head [19]	60.7	78.5	67.8	68.6	81.2
Cascade RCNN [18]	62.7	77.1	67.6	67.9	81.3
Sparse RCNN [47]	58.6	68.1	63.8	69.0	80.1
Dino [48]	61.3	75.6	69.9	69.6	81.2
YOLOV5-L [13]	55.6	71.8	56.6	69.3	81.7
YOLOV5-X [13]	55.5	72.8	58.8	69.8	82.3
YOLOV8-X [14]	51.8	73.6	58.5	69.3	82.5
Ours	63.5	77.2	71.2	70.1	81.3

Feature response maps of different pest images at multiple scales

Figure S3 shows the feature response maps of our method on three different pests at multiple scales. There were differences in the response regions of the features to pest images at different scales. Layer P2 had the strongest response to the characteristics of the target pest, while layer P3 had the weakest response. Additionally, the response location to the target pest differed in layers P4 and P5. As features at different scales can effectively complement each other, suboptimal results that may result from the selection of single-scale features can be avoided by adaptively fusing features from multiple scales.

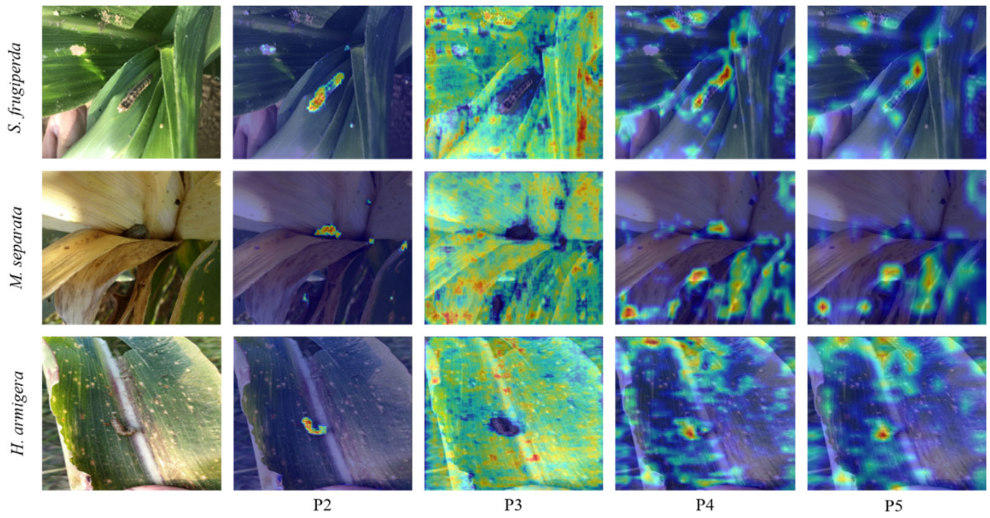

Figure S3. Feature response maps of different pest images at multiple scales. Differences in the feature response regions of the P2-P5 layers.

Identification system

We have developed an online pest identification system based on the proposed method, and users can easily install the application on their Android devices to use it. The system operates through a straightforward three-step process: 1) Image Acquisition: Users can either select an existing pest image from a local photo album or use the camera mode to capture a new pest image. Once the image is selected, the user uploads it to the system. 2) Pest Detection: Upon uploading the image, the system initiates the pest detection process. The uploaded image data is transmitted to the server, which performs model inference for pest detection. 3) Result Display: Once the model inference is completed, the server returns the pest detection result to the client. The user's mobile device then displays the identification results, including the category and number of pests, as well as the habits and control methods of the pests. The specific information can be seen in Figure S4.

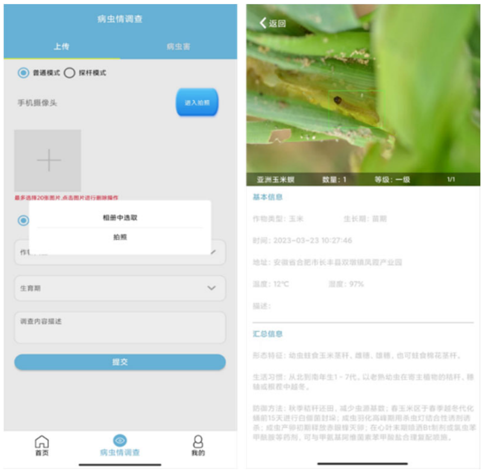

Figure S4. Pest Identification System Client Application.
